# Sequential Organ Failure Assessment (SOFA) score and quick SOFA(qSOFA) predict 30-day mortality in patients with HIV-associated Talaromycosis: A multicenter retrospective cohort study

**DOI:** 10.1371/journal.pntd.0014278

**Published:** 2026-05-05

**Authors:** Guanjing Lang, Shasha Ye, Xingguo Miao, Jiaying Qin, Dairong Xiang, Mengyan Wang, Gong Chen, Feifei Su, Lijun Xu

**Affiliations:** 1 State Key Laboratory for Diagnosis and Treatment of Infectious Diseases, National Clinical Research Center for Infectious Diseases, National Medical Center for Infectious Diseases, Collaborative Innovation Center for Diagnosis and Treatment of Infectious Diseases, Department of Infectious Diseases, The First Affiliated Hospital, Zhejiang University School of Medicine, Hangzhou, China; 2 Yuhang Institute of Medical Science Innovation and Transformation, Hangzhou, China; 3 Department of Infectious Diseases, Wenzhou Central Hospital, Wenzhou, China; 4 Department II of Infectious Diseases, Xixi Hospital of Hangzhou, Zhejiang Chinese Medical University, Hangzhou, China; FIOCRUZ: Fundacao Oswaldo Cruz, BRAZIL

## Abstract

HIV-associated talaromycosis (HAT) is a severe fungal infection for which established severity assessment methods are lacking. The Sequential Organ Failure Assessment (SOFA) and quick SOFA (qSOFA) scores were evaluated in 464 patients with HAT to assess their associations with inflammatory markers, hospital stay, and 30-day mortality. SOFA scores were negatively correlated with blood culture positivity time (r = -0.470, P < 0.001) and positively correlated with IL-6, IL-10, and CRP (all P < 0.001). Patients with fungemia had higher SOFA scores (2.3 ± 2.4 vs. 1.2 ± 0.6, P < 0.001). Mortality increased with qSOFA scores: 8.9% (score 0), 16.5% (score 1), and 55.0% (score ≥2; P < 0.001). For SOFA, mortality was 4.5% (scores 0–1), 6.8% (2–3), 22.0% (4–5), 52.2% (6–7), and 85.7% (≥8; P < 0.001), repectively. Survivors’ SOFA scores improved by day 7 (1.6 ± 1.6 to 1.0 ± 1.4, P < 0.001), while non-survivors worsened by day 7 (4.8 ± 3.4 to 5.1 ± 5.6, P = 0.027) compared to day 0. Among the surviving patients, the hospital stay days were 21.0 (14.0-27.0) for scores 0–1, 22.0 (16.0-29.0) for scores 2–3, 27.0 (20.3-43.5) for scores 4–6 and 29.0 (5.5-38.0) for scores ≥6 (P = 0.005). Multivariate analysis identified qSOFA [adjusted odds ratio (AOR):1.564, P = 0.018], SOFA [AOR:1.533, P = 0.001], and non-amphotericin B deoxycholate (non-AmBd) therapy [AOR:2.732, P = 0.026] were independent predictors of 30-day mortality. SOFA and qSOFA both predicted poor outcomes in patients with HAT. Early diagnosis and preemptive AmBd therapy should be prioritized for patients with HAT who had high SOFA/qSOFA scores.

## Author summary

HIV-associated talaromycosis (HAT) is a life-threatening opportunistic infection in people living with HIV, yet tools for early severity assessment and risk stratification remain limited. In this multicenter retrospective cohort study of 464 patients with HAT, we evaluated the clinical utility of the Sequential Organ Failure Assessment (SOFA) and quick SOFA (qSOFA) scores, and found that both scores were strongly associated with systemic inflammation, fungal dissemination, and clinical outcomes. High SOFA and qSOFA scores at admission correlated with elevated inflammatory markers, shorter blood culture positivity time, and increased disease severity. Importantly, mortality rose sharply with increasing scores, and SOFA demonstrated strong predictive performance for 30-day mortality. Dynamic changes in SOFA scores also reflected responses to treatment, with improvement observed in survivors but deterioration in non-survivors. In addition, lack of amphotericin B deoxycholate (AmBd) induction therapy was independently associated with increased mortality. Our findings suggest that SOFA and qSOFA are practical tools for early bedside risk assessment and monitoring in HAT. Integrating these scoring systems into clinical decision-making may facilitate timely intervention and improve patient outcomes.

## Introduction

*Talaromyces marneffei* (TM) is a fatal fungal pathogen that is endemic in Southeast Asia, northeast India, and southern China [1–3]. HIV-1 infection is the predominant risk factor for disseminated TM infection, with mortality rates up to 20%-30% even under antifungal treatment [4]. Thus, accurate assessment of HIV-associated talaromycosis (HAT) severity and identification of mortality risk factors are vital [5].

The Sequential Organ Failure Assessment (SOFA) score evaluates six organ systems (PaO_2_/FiO_2_, mean arterial pressure, serum creatinine, bilirubin, platelet number, and Glasgow Coma Scale), while the quick SOFA (qSOFA) provides a simplified bedside tool based on respiratory rate, Glasgow Coma Scale, and systolic blood pressure [6]. Both are valuable prognostic tools in critical illness and infectious diseases [7–9].

HAT is typically characterized by disseminated infection, multiple organ dysfunction, and high mortality rates [10]. Given the frequent occurrence of multi-organ dysfunction in patients with HAT, we hypothesized that higher SOFA and qSOFA scores at admission would be associated with greater systemic inflammation, longer hospital stays, and higher 30-day mortality in patients with HAT. Therefore, we performed the present study to explore the associations between SOFA/qSOFA scores, inflammatory markers, hospital stay, and 30-day mortality in a multicenter retrospective cohort study, aiming to establish triage and bedside decision-making system in routine clinical care.

## Materials and methods

### Ethical approval of the study protocol

This study protocol was conducted in accordance with the 1975 Declaration of Helsinki and was approved by the Ethics Committee of the First Affiliated Hospital, School of Medicine, Zhejiang University (Hangzhou, China) (No. IIT20210532A). The ethical Committee waived the consent of patient. All data were analyzed anonymously.

### Patient selection

From January 2018 to December 2024, 575 patients with HAT from three hospitals in China were retrospectively analyzed. Patients who met the following criteria were included: (1) seropositive HIV status, (2) age ≥ 18 years, and (3) laboratory confirmation of TM infection. The exclusion criteria were: (1) prior antifungal treatment; (2) prior antiretroviral therapy; (3) age < 18 years; (4) treatment abandonment; and (5) absence of necessary laboratory and clinical records. [11]. Of those patients, 81 on antiviral treatment, 17 ceased treatments, 9 with malignances and 4 under 18 years were excluded from the cohort. A total of 464 patients were enrolled in the present multicenter retrospective cohort study.

### Routine blood test, biochemical test and T lymphocyte subgroup measurements

Blood samples were drawn immediately upon admission. All samples were processed and examined immediately in the central laboratory of each hospital. The complete blood count was measured in Hematology analyzers (Beckman Coulter, CA, USA) and biochemical tests were measured using an Auto-biochemical Analyzer (Beckman Coulter, CA, USA). The number of CD4+ and CD8 + T cells were measured using flow cytometry (BD biosciences, NJ, USA).

### Chemokine and cytokine assay

Interleukin (IL)-4, IL-6, IL-10, IL-17, tumor necrosis factor-alpha (TNF-α), and interferon-gamma (IFN-γ) levels were quantified using flow cytometry. The detailed procedures were performed according to the manual for the Cytokine FCM kit (BD biosciences, NJ, USA) (S1 File).

### Diagnosis criteria

#### Talaromycosis and fungemia.

The diagnosis of Talaromycosis was established based on any of the following criteria: (1) positive TM culture from peripheral blood, bone marrow, alveolar lavage, or other specimens; (2) detection of typical yeast cells (2–3 µm in diameter with oval, round, or sausage-like shapes) on tissue sections stained by periodic acid-Schiff or Wright’s stain; and (3) identification of TM DNA sequences via metagenomics next-generation sequencing (mNGS) from body fluids or tissues [11,12].

Fungemia was defined as the detection of TM in peripheral blood by either culture or mNGS in patient with HAT presenting with recurrent fever, rash, severe anemia, splenomegaly, or lymphadenopathy [13].

#### SOFA and qSOFA score calculation.

The calculation of SOFA score and qSOFA was performed according to the Sepsis-3 definition [6]. The detailed score point can be referenced to S2 File.

### Antifungal therapy

Antifungal therapy was administered to all patients immediately after the diagnosis of talaromycosis. The initial induction therapy involved intravenous amphotericin B deoxycholate (AmBd) (0.7 mg/kg/day), Voriconazole (8 mg/kg/day) (divided into two doses, with a loading dose of 12 mg/kg/day on the first day), or itraconazole (400 mg/day) (divided into two doses) for 2 weeks, followed by itraconazole (400 mg daily for 10 weeks) as consolidation therapy, and then maintenance therapy with itraconazole (200 mg daily) until CD4 counts exceed 100 cells/mm³ for at least 6 months. The initial induction therapy was performed according to clinical guidelines and available antifungal agents [14,15].

### Follow-up and outcome of patients

In China, most PLWH were registered in the electronic system of the National Free Antiretroviral Treatment Program (NFATP). The information of demographics, laboratory test results, clinical signs and symptoms at admission recorded in both electronic medical records system (EMRS) of each hospital and NFATP. NFATP system provided a real-time and lifelong data information of patients on ART regimen, CD4 count, HIV-RNA load and survival/death. In our study, the primary outcome was defined as overall 30-day mortality [16].

### Sample size calculation

A total of 61 deaths (events) occurred among 464 patients. Multivariate Cox regression model was used to identify the risk factors related to 30-day mortality and 12 covariates were taken into the model. According to the commonly accepted rule of at least 10-time cases for per variable in Cox regression models, at least 120 cases should be reliably included in the multivariate model. In this study, the number of patients included in the final multivariate model far exceed this threshold, ensuring the robustness and stability of the regression estimates.

### Statistical analyses

Continuous normally distributed variables were presented as the means ± standard deviations (SD) or medians (interquartile ranges, IQRs) and compared by Student’s *t*-test or nonparametric test. Categorical variables were presented as the number of cases (percentage) and compared by *χ2* analysis or Fisher’s exact test. Receiver operating characteristic (ROC) curve with the Youden index was employed to assess diagnostic performance. The diagnostic performance of SOFA and qSOFA scores was evaluated by area under curve (AUC). Associations of the serum cytokine/chemokine levels with SOFA and qSOFA scores were analyzed by principal component analysis (PCA). The serum cytokine/chemokine concentrations were log_10_-transformed for analysis. Univariate Cox proportional regression models were utilized to identify risk factors for poor prognosis. Covariates with P < 0.15 in the univariate model were included in the multivariate Cox proportional regression model to determine independent risk factors for poor prognosis. Statistical significance was set at P < 0.05 (two-tailed). The data were analyzed using SPSS version 26.0 (IBM, NY, USA) and GraphPad Prism software 9 (GraphPad Software, CA, USA).

## Results

### Demographic information and clinical characteristics

Among 464 patients (88.6% male, mean age 38.3 ± 12.0 years, BMI 19.9 ± 3.5 kg/m^2^), median CD4 count was 10.0 (4.0-24.3) cells/μL. There were 305 (65.7%) patients with qSOFA score of 0, 139 (30.0%) with qSOFA score of 1, 18 (3.9%) with qSOFA score of 2 and 2 (0.4%) with qSOFA score of 3. Meanwhile, there were 223 (48.1%) patients with SOFA score of 0–1, 147 (31.7%) with SOFA score of 2–3, 50 (10.8%) with SOFA score of 4–5, 23 (5.0%) with SOFA score of 6–7 and 21 (4.5%) with SOFA score ≥8.

The overall 30-day mortality rate was 13.1% (61/464). The deceased patients were older (years; 42.1 ± 13.4 vs. 37.8 ± 11.7, *P* = 0.009), had lower level of BMI (kg/m^2^; 18.8 ± 3.1 vs 20.0 ± 3.5, *P* = 0.018), hemoglobin (g/L; 90.3 ± 22.7 vs. 98.5 ± 29.7, P = 0.005) and platelet [×10^9^/L; 54.0 (25.0 - 114.5) vs. 115.0 (65.8 - 194.0), P < 0.001], but had higher level of white blood count (WBC)[×10^9^/L; 4.3 (2.9 - 6.1) vs. 3.5 (2.3 - 5.1), *P* = 0.023], aspartate aminotransferase (AST) [U/L; 83.0 (33.0 - 181.0), vs. 51.5 (28.0 - 98.0), *P* < 0.001], LDH [U/L; 638.0(408.0 - 1302.0) vs. 400.0 (264.0 - 638.0), *P* < 0.001] and C-reactive protein (CRP) [(mg/L;74.5(36.6-127.0) vs. 55.4(26.0-92.0), *P* = 0.016] than surviving patients. The detailed characteristics of patients are shown in Table 1.

**Table 1:** The demographic characteristics of patients with HAT (n = 464).

All patients	Results (n = 464)	Survival (n = 403)	death (n = 61)	P value
**Sex** [male (%)]	411 (88.6)	358 (88.9)	53 (86.9)	0.666
**Age** (years)	38.3 ± 12.0	37.8 ± 11.7	42.1 ± 13.4	0.009
**BMI** (kg/m^2^)	19.9 ± 3.5	20.0 ± 3.5	18.8 ± 3.1	0.018
**Laboratory tests**				
CD4+ (cells/μL)	10.0 (4.0-24.3)	10.0 (4.0-24.0)	10.0 (4.8-34.3)	0.534
WBC(×10^9^/L)	3.6(2.4-5.2)	3.5 (2.3-5.1)	4.3(2.9-6.1)	0.010
Platelet (*×*10^9^/L)	109.0 (56.0-181.0)	115.0(65.8-194.0)	54.0(25.0-114.5)	< 0.001
Hemoglobin (g/L)	97.4 ± 21.2	98.5 ± 29.7	90.3 ± 22.7	0.005
Albumin (g/L)	28.0 ± 6.3	28.6 ± 6.3	24.2 ± 5.2	< 0.001
ALT(U/L)	17.0 (8.0-44.0)	17.0 (0.8-44.0)	16.0 (0.7-44.8)	0.976
AST(U/L)	55.0 (28.0-105)	51.5 (28.0-98.0)	83.0 (33.0-181.0)	0.003
TB(μmol/L)	9.7(6.8-15.3)	9.3(6.6-14.8)	12.7(8.6-30.6)	< 0.001
Creatinine (µmol/L)	66.0(56.0-78.0)	65.5(56.0-76.8)	70.0(57.5-136.0)	0.018
Urea (mmol/L)	4.3(3.3-6.0)	4.1(3.2-5.4)	7.5(4.7-13.0)	< 0.001
LDH (U/L)	429.0 (277.8-679.0)	400.0 (264.0-638.0)	638.0 (408.0-1302.0)	< 0.001
CRP (mg/L)	57.2 (26.4-99.2)	55.4 (26.0-92.0)	74.5(36.6-127.0)	0.016
**Major comorbidities [n(%)]**				
Tuberculosis	15 (3.2)	15 (3.7)	0 (0)	0.237
Non-Tuberculous Mycobacteria	15 (3.2)	14 (3.5)	1 (1.6)	0.705
Pneumocystis pneumonia	50 (10.8)	42 (10.4)	8 (13.1)	0.527
Hepatitis B virus	31 (6.7)	27 (6.7)	4 (6.6)	0.967
Hepatitis C virus	1 (0.2)	1 (0.2)	0 (0)	–
Cryptococcus	14 (3.0)	13 (3.2)	1 (1.6)	1.000
Malignances	12 (2.6)	11 (2.7)	1 (1.6)	1.000
**qSOFA**	0 (0-1)	0(0-1)	1(0-1)	< 0.001
0	305(65.7)	278 (70.0)	27 (44.2)	< 0.001
1	139 (30.0)	116 (28.8)	23 (37.7)	
2	18(3.9)	9 (2.2)	9 (14.8)	
3	2(0.4)	0 (0)	2 (3.3)	
**SOFA**	2 (0-3)	1(0-3)	5.0(3-8)	< 0.001
**0-1**	223 (48.1)	213(52.9)	10 (16.4)	< 0.001
**2-3**	147(31.7)	137(34.0)	10(16.4)	
**4-5**	50(10.8)	39(9.7)	11(18.0)	
**6-7**	23(5.0)	11(2.7)	12(19.7)	
**≥ 8**	21(4.5)	3(0.7)	18(29.5)	
**Initial induction therapy [n(%)]**				
AmBd	117 (25.2)	110(27.3)	7 (11.5)	0.008
Non-AmBd	347 (74.8)	293(72.7)	54 (88.5)	
**30-day mortality [n(%)]**	61 (13.1)	0	61	–

Abbreviations: BMI, body mass index; WBC, white blood cell; ALT, alanine transaminase; AST, aspartate aminotransferase; TB, total bilirubin; CRP, C-reactive protein; LDH, lactic dehydrogenase; SOFA, Sequential Organ Failure Assessment; qSOFA, quick SOFA. AmBd, amphotericin B deoxycholate.

### Blood culture result was associated with SOFA/qSOFA scores

**Fig 1:**
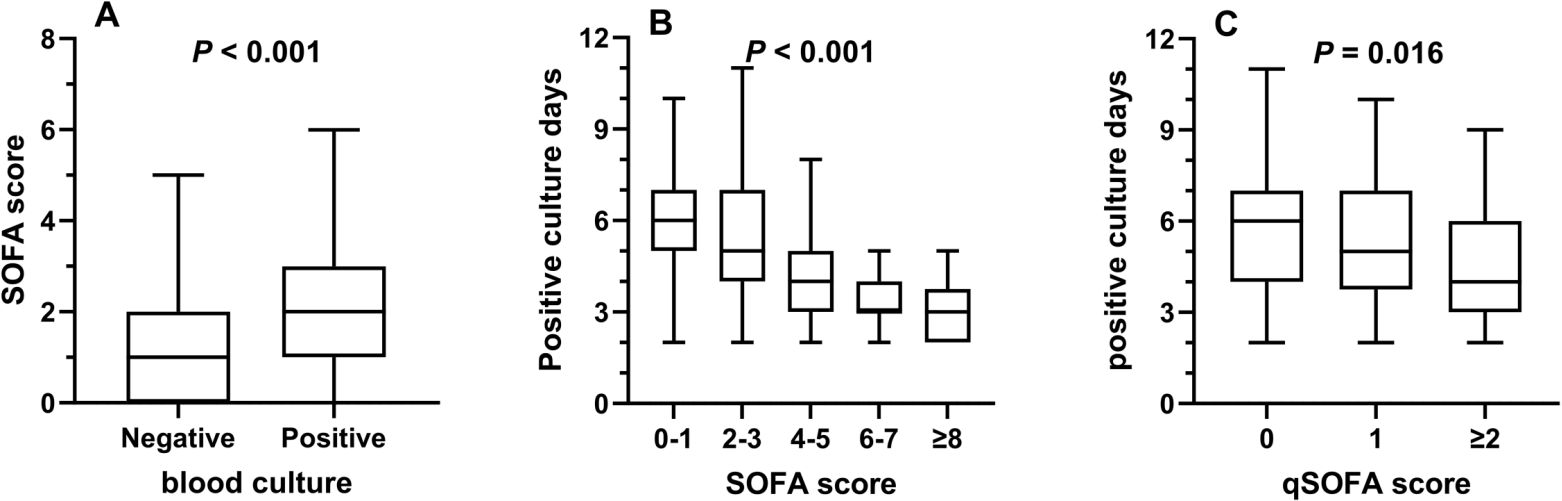
SOFA/qSOFA scores and culture positivity time (CPT). **A.** Patients with positive blood culture had high SOFA scores (*P* < 0.001); **B.** Patients with higher SOFA scores exhibited significantly shorter culture positive time (CPT) (*P* < 0.001). **(c)** A high qSOFA score was negatively associated with CPT (P = 0.016).

Among those patients with HAT, 368 (79.3%) were diagnosed via positive blood culture, 21 (4.5%) via positive blood mNGS result, and 3 (0.6%) via both positive blood culture and NGS result, 25 (5.4%) via positive mNGS result from bronchoalveolar lavage fluid (BALF), 30 (6.5%) via positive histopathology, and 45 (9.7%) by positive bone marrow culture. A total of 392 (84.5%) patients had fungemia and while 72 (15.5%) did not have fungemia. The SOFA score was 2.0 (1.0–3.0) in those with fungemia and 1.0 (0–2.0) in those without (*P* < 0.001) (Fig 1A), meanwhile, qSOFA score was 0.5 (0–1.0) in patients with fungemia and 0 for those without (*P* < 0.001), respectively.

Among those with fungemia, 207/392 (52.8%) had recorded the culture positivity time (CPT), whereas 185 (47.2%) did not have CPT. The CPT was 6.0 (5.0 -7.0) days for patients with a SOFA score of 0–1, 5.0 (4.0-7.0) for a score of 2–3, 4.0 (3.0-5.0) for a score of 4–5, 3.0 (3.0-4.0) days for a score of 6–7, and 3.0 (2.0-3.8) days for a score ≥8.0 (*P* < 0.001) (Fig 1B). Similarly, the CPT was 6.0 (4.0–7.0) days for patients with a qSOFA of 0, 5.0 (3.8–7.0) days for patients with qSOFA score of 1, and 4.0 (3.0–6.0) days for patients with qSOFA score ≥ 2.0 (*P* = 0.016) (Fig 1C). Both SOFA and qSOFA scores were inversely associated with CTP (r = -0.470, *P* < 0.001; r = -0.196, *P* = 0.004, respectively).

### qSOFA/SOFA scores associated with severity of infection

**Fig 2:**
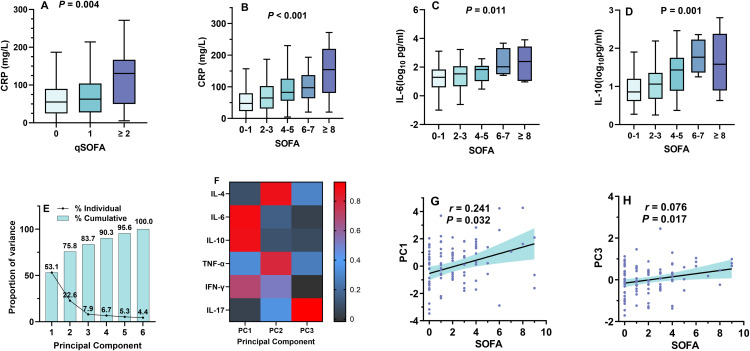
SOFA/qSOFA scores and inflammation. The level of C-reactive protein (CRP) was increased with qSOFA **(A)** and SOFA score **(B)**; the level of serum IL-6 **(C)** and IL-10 **(D)** was higher in patients with had high SOFA score; Scree plot illustrating 3 principal components (PC) 1, 2 and 3 responsible for 83.7% events **(E)**; Heatmap indicated that PC1 was primarily driven by IL-6 and IL-10, PC2 by IL-4 and TNF-α, and PC3 by IL-17 **(F)**; PC1(**G**) and PC3 (**H**) was both positive with SOFA.

The relationship between qSOFA/SOFA scores and serum CRP levels was analyzed. The level of CRP was 55.1(24.8-90.0) mg/L in patients with a qSOFA score of 0, 62.5(27.7-104.2) mg/L in those with a qSOFA score of 1 and 130.7 (49.8-167.0) mg/L in those with a qSOFA score ≥ 2.0 (*P* = 0.004) (Fig 2A). Similarly, the level of CRP was 47.5 (23.2–79.5) mg/L for patients with a SOFA score of 0–1, 64.7 (31.0–102.1) mg/L for scores of 2–3, 82.4 (55.8–125.5) mg/L for scores of 4–5, 97.0 (63.7–137.0) mg/L for scores of 6–7, and 154.2 (80.3–219.8) mg/L for scores ≥ 8 (*P* < 0.001) (Fig 2B).

Serum level of interleukin (IL)-4, IL-6, IL-10, IL-17, tumor necrosis factor-α (TNF-α), and interferon-γ (IFN-γ) were measured in 122 patients with preserved serum sample available. The serum concentration was -0.1 ± 0.6 (log_10_ pg/mL) for IL-4, 1.4 ± 0.6 (log_10_ pg/mL) for IL-6, 1.1 ± 0.6 (log_10_ pg/mL) for IL-10, 0.0 ± 0.0 (log_10_ pg/mL) for IL-17, 0.2 ± 0.5 (log_10_ pg/mL) for TNF-α and 0.8 ± 0.8(log_10_ pg/mL) for IFN-γ. The concentration of IL-6 was 1.3(0.6-1.8) (log_10_ pg/mL)for SOFA score of 0–1, 1.5(0.7-2.1) (log_10_ pg/mL) for SOFA score of 2–3, 1.8(1.0-2.1) (log_10_ pg/mL) for SOFA score of 4–5, 2.0(1.5-3.3) (log_10_ pg/mL)for SOFA score of 6–7 and 2.4(1.0-3.4) (log_10_ pg/mL)for SOFA score ≥8 (*P* = 0.011) (Fig 2C). Similarly, the concentration of IL-10 was 0.9(0.6-1.2) (log_10_ pg/mL) for SOFA score of 0–1, 1.1(0.7-1.4) (log_10_ pg/mL) for SOFA score of 2–3, 1.4(0.9-1.8) (log_10_ pg/mL) for SOFA score of 4–5, 1.8(1.4-2.3) (log_10_ pg/mL) for SOFA score of 6–7 and 1.6(0.9-2.4) (log_10_ pg/mL) for SOFA score ≥8 (*P* = 0.001) (Fig 2D). No significant association was observed between other cytokines (IL-4, IL-17, TNF-α and IFN-γ) and SOFA/qSOFA scores.

The principal component analysis (PCA) revealed that the first three components (PC1, PC2, and PC3) explained 83.7% of the total variance in cytokine levels, with PC1 accounting for 53.1%, PC2 for 22.6%, and PC3 for 7.9% (Fig 2E). PC1 was primarily driven by IL-6 and IL-10, while PC2 was mainly driven IL-4 and TNF-α. PC3 was mainly driven by IL-17 (Fig 2F). Best-fit linear regression indicated that SOFA score was correlated with PC1(*r* = 0.241, *P* = 0.032) (Fig 2G) and PC3 (*r* = 0.076, *P* = 0.017) (Fig 2H).

### SOFA/qSOFA scores and hospital stay-days among surviving patients

**Fig 3:**
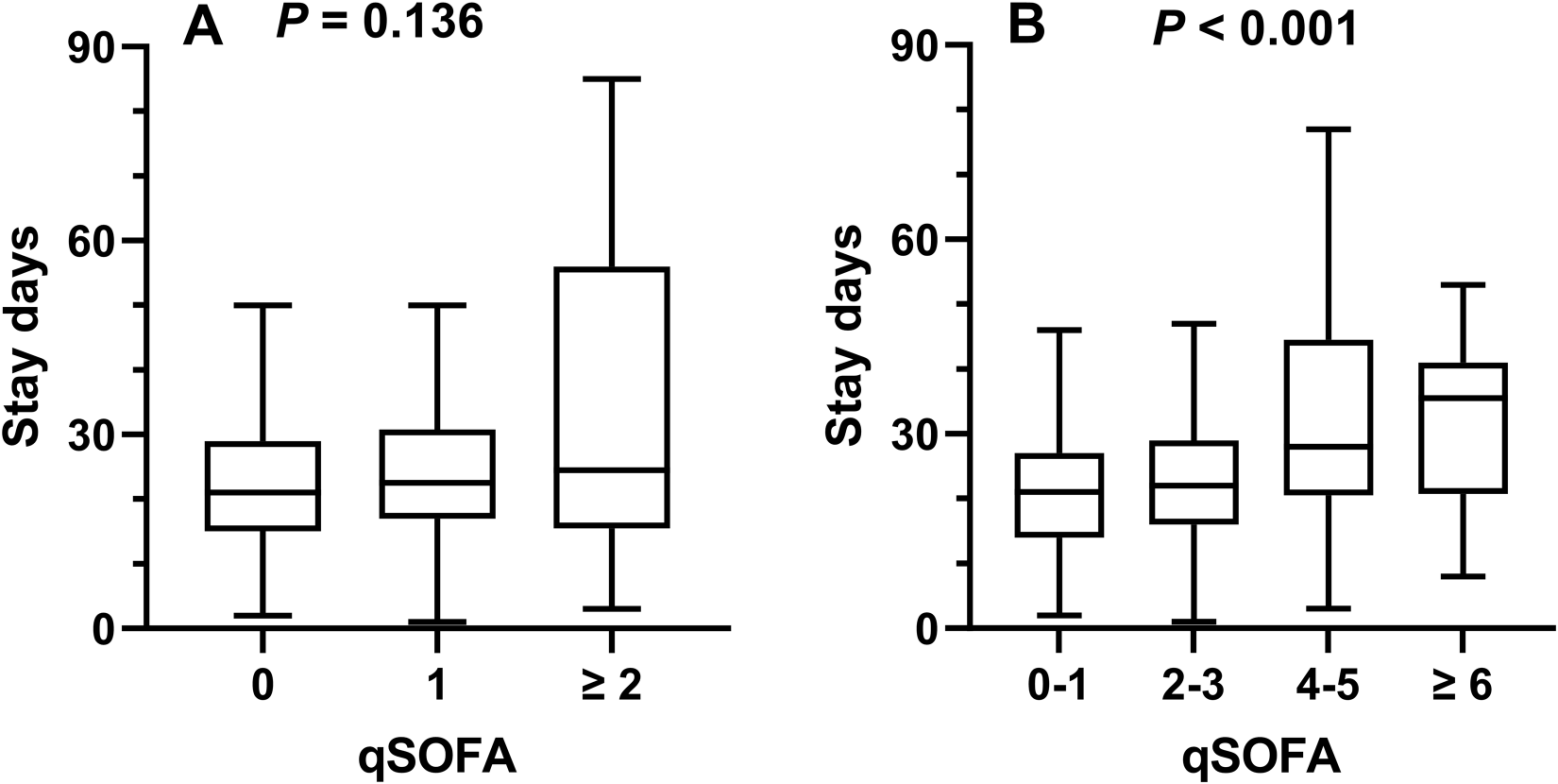
SOFA/qSOFA scores and hospital stay duration. The hospital stay day in patients with different qSOFA **(A)** and SOFA score **(B)** in surviving patients.

We analyzed the relationship between the length of hospital stay and SOFA/qSOFA scores among patients who survived during hospitalization. The stay-days were 21.0(15.0-29.0) days for patients with a qSOFA score of 0, 22.5(17.0-30.8) days for those with a qSOFA score of 1 and 24.5(16.5-56.0) days for those with a qSOFA score ≥ 2.0 (*P* = 0.136) **(**Fig 3A). Meanwhile, the stay-days were 21.0(14.0-27.0) for patients with a SOFA score of 0–1, 22.0(16.0-29.0) for those with a SOFA score of 2–3, 27.0 (20.3-43.5) days for those with SOFA score of 4–5, and 29.0 (5.5-38.0) days for those with a SOFA score ≥ 6 (*P* = 0.005) (Fig 3B).

### SOFA, qSOFA scores and 30-day mortality

**Fig 4:**
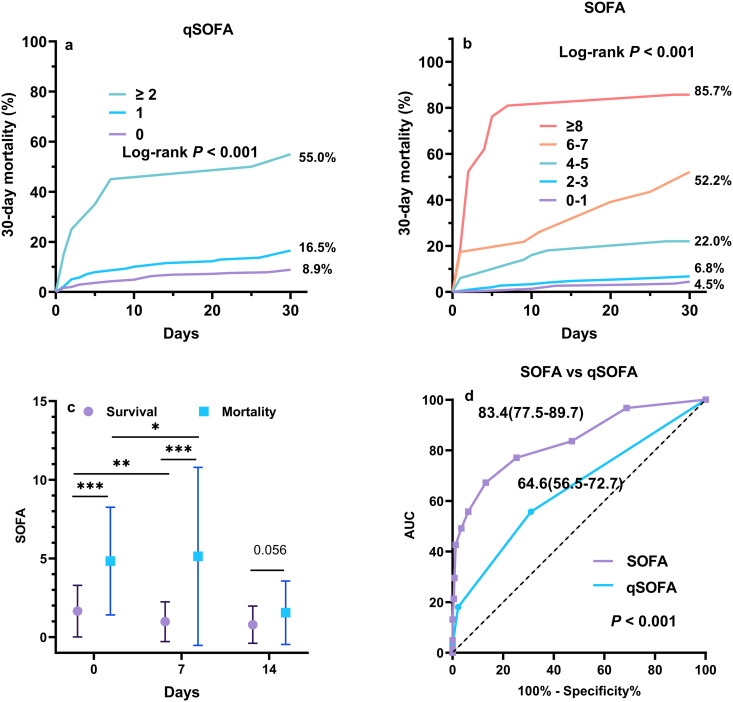
SOFA/qSOFA scores and 30-day mortality. **A.** 30-day mortality in patients with different qSOFA scores; **B.** 30-day mortality in patients with different SOFA scores; **C.** Deceased patients had significantly higher SOFA at day 0 and day 7 than those survival patients (*P* < 0.001). **D.** SOFA score demonstrated significantly greater predictive accuracy for 30-day mortality compared to qSOFA score (*P* < 0.001). *: *P* < 0.05, **: *P* < 0.01, ***: *P* < 0.001.

The 30-day cumulative mortality rate was 8.9% for patients with qSOFA score of 0, 16.5% for those with qSOFA score of 1, and 55.0% for those with qSOFA score of ≥2 at admission (Log-rank *P* < 0.0001) (Fig 4A).

The 30-day mortality rate was 4.5% for those with SOFA score of 0–1, 6.8% for those with SOFA score of 2–3, 22.0% for those with SOFA score of 4–5, 52.2% for those with SOFA score of 6–7, and 85.7% for those with SOFA score ≥ 8 (Log-rank P < 0.001) (Fig 4B), respectively.

The mean SOFA score was 1.6 ± 1.6 (day 0) in surviving patients and 4.8 ± 3.4 (day 0) in non-survival patients at admission (*P* < 0.001), More importantly, the SOFA score of 1.6 ± 1.6 at day 0 was decreased into 1.0 ± 1.4 at day 7 in survivors (*P* = 0.007) after one week of antifungal therapy. However, the SOFA score of 4.8 ± 3.4 at day 0 was increased up to 5.1 ± 5.6 at day 7 (*P* = 0.027) in non-survival patients after one week of antifungal therapy (Fig 4C)

The diagnostic performance of SOFA and qSOFA scores were compared. The AUC was 0.646(95%CI:0.600-0.689) for qSOFA and 0.836(95 CI:0.799-0.868) for SOFA (*P* < 0.001) (Fig 4D).

### Effects of induction therapy on 30-day overall mortality of patients with different SOFA/qSOFA scores

**Fig 5:**
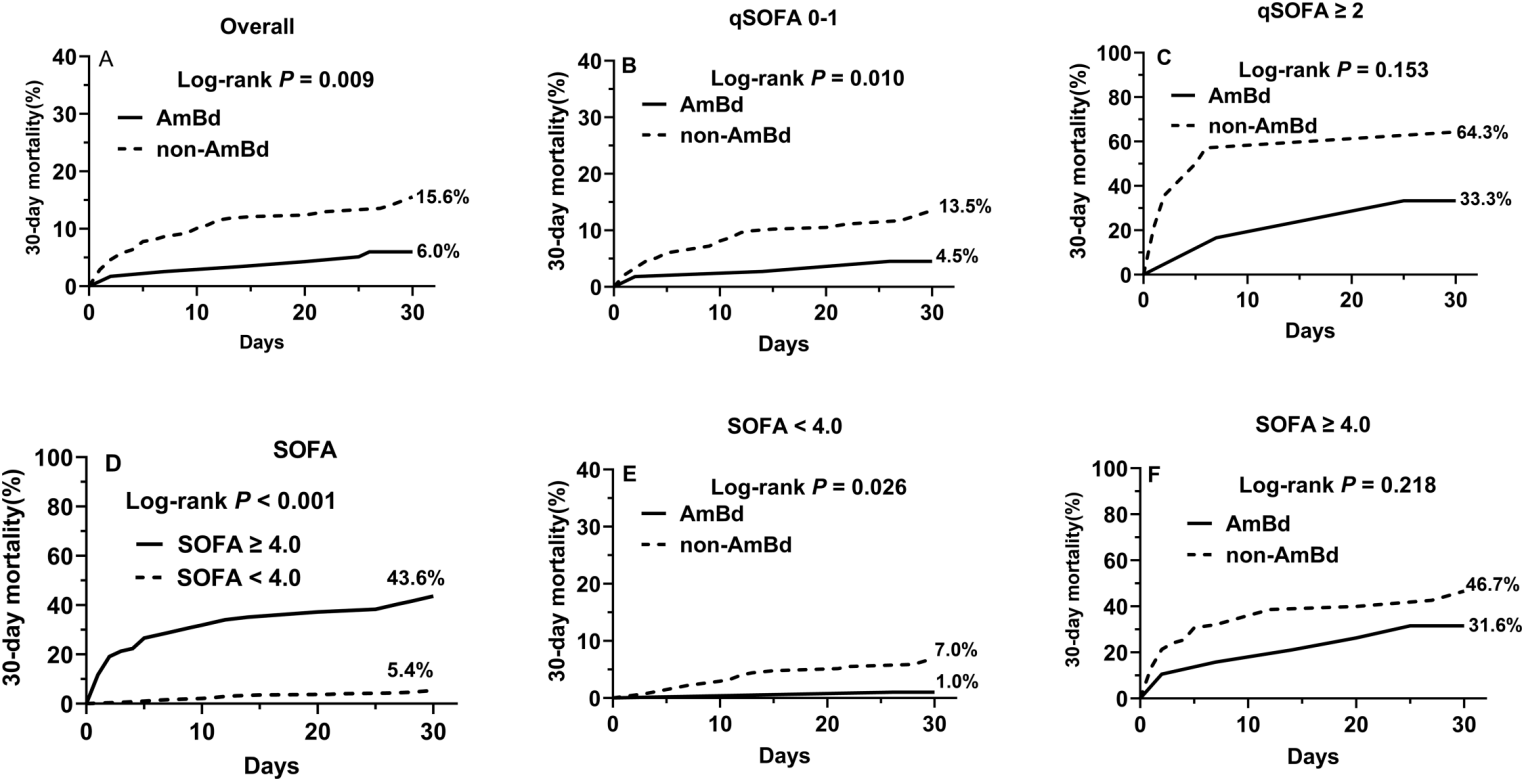
Impact of induction therapy on outcomes across SOFA/qSOFA score subgroups. **A.** Overall 30-day mortality rates in patients receiving AmBd versus non-AmBd induction therapy; **B-C:** Effects of AmBd or non-AmBd therapy on 30-day mortality in patients with qSOFA of 0-1 and qSOFA score ≥2 group. **D.** 30-day mortality in patients with SOFA < 4.0 and ≥4.0. **E-F:** Effects of AmBd or non-AmBd therapy on 30-day mortality in patients with SOFA < 4.0 and SOFA score ≥ 4.0.

There were 25.2% (117/464) patients treated with AmBd induction therapy and 74.8 (347/464) treated with non-AmBd induction therapy. The overall 30-day mortality was 6.0% in AmBd induction therapy and 15.6% in non-AmB induction therapy (Log-rank *P* = 0.009) (Fig 5A).

The overall 30-day mortality was 4.5% for AmBd treatment and 13.5% for non-AmBd treatment in the patients with qSOFA score of 0–1 (Log-rank *P* = 0.010) (Fig 5B), 33.3% for AmBd treatment and 64.3% for non-AmBd treatment in the patients with qSOFA score ≥ 2.0 (Log-rank *P* = 0.153) (Fig 5C).

Youden index suggested SOFA score ≥ 4.0 was the cutoff point for prediction of 30-day mortality. The overall 30-day mortality was 5.4% in those with SOFA score < 4.0 and 43.6% in those with SOFA score ≥ 4.0 (Log-rank P < 0.001) (Fig 5D). Furthermore, the overall 30-day mortality was 1.0% in AmBd treatment and 7.0% in non-AmBd group among patients with SOFA score < 4.0 (Log-rank *P* = 0.026) (Fig 5E), 31.6% in AmBd group and 46.7% in non-AmBd group among patients with SOFA ≥ 4.0 (Log-rank *P* = 0.218) (Fig 5F).

### Factors influencing the 30-day mortality of HAT

Univariate Cox analysis was used to analyze 12 factors, including sex, age, BMI, CD4 count, WBC, hemoglobin, albumin, CRP, LDH, AmB induction, SOFA, qSOFA scores and fungemia. The results revealed that age [odd ratio (OR) (95% confidential interval):1.027 (1.007-1.048), *P* = 0.009], BMI [OR:0.914 (0.854-0.979), *P* = 0.010], WBC [OR: 1.136 (1.063-1.214), *P* < 0.001], hemoglobin [OR: 0.983 (0.971-0.995), P = 0.005], albumin [OR: 0.894 (0.856-0.993), *P* < 0.001], C-reactive protein [OR: 1.008 (1.004 - 1.012), *P* < 0.001], AST [OR:1.002(1.002-1.003), *P* < 0.001], LDH [OR: 1.000 (1.000-1.000), *P* < 0.001], qSOFA score [OR: 2.578 (1.853-3.586), *P* < 0.001], SOFA score [OR: 1.616 (1.491-1.751), *P* = 0.001] and non-AmBd induction [OR: 2.744 (1.248-6.030), *P* = 0.012] were risk factors related to overall 30-day mortality. Additionally, fungemia [OR: 2.158(0.865-5.388), *P* = 0.099] was close to overall 30-day mortality.

In the multivariate Cox proportional risk model, our data showed that low BMI [adjusted OR (AOR):0.913 (0.837 - 0.995), *P* = 0.037], high WBC [AOR: 1.090 (1.015 - 1.171), *P* = 0.018], high LDH [AOR: 1.000 (1.000 - 1.001), *P* = 0.021], high qSOFA score [AOR: 1.564 (1.080 - 2.266), *P* = 0.018], high SOFA score [AOR: 1.533(1.362 - 1.726), *P* < 0.001] and non-AmBd induction [AOR: 2.732(1.128-6.618), P = 0.026] were independent risk factors related to overall 30-day mortality (Table 2).

**Table 2:** Risk factors for 30-day mortality in patients with HAT in univariate/multivariate Cox proportional risk models.

Factor	Univariate		Multivariate	
	OR (95% CI)	P	OR (95% CI)	P
**Age (year)**	1.027 (1.007-1.048)	0.009	1.014(0.991-1.037)	0.241
**BMI (kg/m**^**2**^)	0.914 (0.854-0.979)	0.010	0.913(0.837-0.995)	0.037
**CD4 count(cells/mm**^**3**^)	1.002(0.996-1.008)	0.584	–	–
**WBC (×10** ^**9**^ **/L)**	1.136 (1.063-1.214)	<0.001	1.090(1.015-1.171)	0.018
**Hemoglobin (g/L)**	0.983 (0.971-0.995)	0.005	0.992(0.977-1.008)	0.334
**Albumin (g/L)**	0.894 (0.856-0.993)	<0.001	1.043(0.986-1.103)	0.145
**CRP (mg/L)**	1.008 (1.004-1.012)	<0.001	0.997(0.992-1.002)	0.291
**AST(U/L)**	1.002(1.002-1.003)	<0.001	1.001(0.999-1.003)	0.140
**LDH (U/L)**	1.000 (1.000-1.000)	<0.001	1.000(1.000-1.001)	0.021
**Fungemia**	2.158(0.865-5.388)	0.099	1.467(0.543-3.966)	0.450
**qSOFA**	2.578(1.853-3.586)	<0.001	1.564(1.080-2.266)	0.018
SOFA	1.616(1.491-1.751)	<0.001	1.533(1.362-1.726)	<0.001
**Non-AmB induction**	2.7 44(1.248-6.030)	0.012	2.732(1.128-6.618)	0.026

OR, odds ratio; BMI, body mass index; WBC, white blood cell; AST, aspartate aminotransferase; CRP, C-reactive protein; LDH, lactic dehydrogenase; SOFA:Sequential Organ Failure Assessment; qSOFA: quick SOFA.

## Discussion

HAT is a severe fungal infection, particularly in advanced stages of HIV. Early and accurate assessment of illness severity is crucial for guiding therapeutic decisions and improving outcomes. Despite its relevance, the application of the SOFA score in HIV/AIDS and opportunistic co-infections remains limited [17–19]. In this study, we validated SOFA and qSOFA in patients with HAT, a population where these scores have been insufficiently studied, and found that 1) they are reliable tools for evaluating disease severity in HAT; 2) organ dysfunction is closely associated with fungal dissemination and inflammation in patients with HAT; 3) SOFA and qSOFA are not only hallmarks of severity of HAT, but also early response markers of antifungal treatment; 4) an early and aggressive therapy (AmBd) should be started in patients with high scores for whom early intervention may be life-saving and 5) integration of scoring into clinical triage and management pathways is pivotal for medical care of patients with HAT.

Previous studies have shown that SOFA score correlates with inflammatory markers such as IL-6 and CRPin the critically ill patients, including those with COVID-19 or critical ICU patients [20,21]. Consistent with these results, our study found significant associations between inflammatory cytokines and SOFA/qSOFA scores. The positive correlation between CRP and both SOFA and qSOFA scores reflects the well-established role of CRP as a systemic marker of inflammation and infection. Higher CRP levels in patients with higher SOFA/qSOFA scores indicate that the inflammatory response is a key driver of disease severity and prognosis. The cytokine analysis revealed that IL-6, a pro-inflammatory cytokine, and IL-10, an anti-inflammatory cytokine, has been linked to severe fungal infection and organ dysfunction, highlighting the complex balance between pro- and anti-inflammatory responses in critically ill patients. The principal component analysis (PCA) further elucidated that IL-6 and IL-10 contribute most significantly to the variation in inflammatory profiles, which may offer a new avenue for identifying patients at higher risk for poor outcomes. The study also confirmed the relationship between blood culture positivity and qSOFA/SOFA scores, offering deeper insights into the clinical trajectory of patients with HAT. Patients with positive blood cultures, a hallmark of fungal dissemination, had significantly higher SOFA/qSOFA scores compared to those with negative blood cultures. This finding reinforces the notion that elevated SOFA scores reflect the severity of disseminated infection. Furthermore, the negative correlation between blood culture positivity time (CPT) and SOFA/qSOFA scores suggests that more severe fungemia is associated with greater organ dysfunction. These findings highlight organ dysfunction is closely related to inflammation and fungal dissemination in patients with HAT.

Both SOFA and qSOFA scores significantly affect clinical outcomes in patients with HAT. Patients with higher SOFA scores have notably longer hospital stays compared to patients with lower scores, indicating that disease severity profoundly impacts recovery among survivors. Furthermore, mortality of patients significantly is increased with rising qSOFA scores (from 8.9% with qSOFA score = 0 to 55.0% with qSOFA score ≥2) and SOFA scores (from 4.5% with SOFA score 0–1 to 85.7% with SOFA score ≥8). Our study indicates a significant correlation between a qSOFA score ≥1 and high overall 30-day mortality rates in patients with HAT, highlighting qSOFA score as an effective, simple and rapid bedside tool for quickly identifying high-risk patients upon admission [22].

More importantly, the SOFA score provides a comprehensive association with inflammatory marker, hospital length of stay, fungal burden and mortality in patients with HAT. In addition, sequential SOFA score closely reflects patient mortality; survivors exhibited significant declines in SOFA score at day 7 compared to day 0, whereas non-survivors show deterioration in SOFA score at day 7. These findings suggest SOFA/qSOFA not only serve as indicators of severity of disease, but also as early indicator of response to effective antifungal therapy.

The effect of antifungal therapy, particularly AmBd induction, is a crucial aspect of this study. The univariate and multivariate Cox regression analyses identify independent risk factors for 30-day mortality, including low BMI, high level of WBC count, LDH, SOFA and qSOFA scores, and non-AmBd induction therapy. Higher SOFA and qSOFA scores are both independently associated with increased overall 30-day mortality, confirming their value as prognostic markers in this population. More importantly, non-AmBd induction therapy is an independent risk factor for mortality, suggesting that alternative antifungal therapies may not be as effective in reducing mortality in severe HAT cases, especially in those with high SOFA and qSOFA scores. Our recent study indicates that AmB is superior to Voriconazole as initial therapy on treatment of HAT with fungemia [13]. These findings reinforce the current therapeutic guidelines recommending AmBd as the gold standard induction therapy for *Talaromyces marneffei* sepsis [23]. Given these findings, our data strongly support that early empirical and aggressive antifungal therapy (AmBd) should be preferred for patients with HAT with high SOFA and qSOFA scores.

Our study has some limitations: 1) The retrospective design introduces the potential for residual confounding. For example, the small size of patients with qSOFA ≥2 (n = 20) and patients who were antifungal-experienced treatment were excluded,which might produce some biases for study; 2) The impact of HIV-RNA on patient outcome is not assessed in our study. This is because HIV-RNA test is not included in NFATP during our study period; 3) Although many researches have revealed a close association between SOFA/qSOFA scores and ICU admission rates, this relationship was not observed in our study, due to the limited availability of ICU resources for PLWH in China; 4) Direct relationship between fungal burden (such as Colony-Forming Unit or Talaromyces marneffei DNA level in blood) and SOFA score was not observed in our study.

In conclusion, this study demonstrates that the qSOFA score serves as a simple and rapid tool for identifying high-risk patients with HAT, while the SOFA score provides a comprehensive evaluation of disease severity, clinical outcome and therapeutic strategies. AmBd induction therapy should be promptly initiated in patients with suspected talaromycosis with high qSOFA or SOFA scores, even in those without blood culture confirmation, to improve clinical outcomes. Integration of scoring into clinical triage and management pathways is necessary for patients with HAT during medical practice.

## Supporting information

S1 File Process of cytokine and chemokine assay by FCM. (DOCX)

S2 File The criteria for assessment of the Sequential Organ Failure Assessment (SOFA) score and qSOFA scores. (DOCX)
