# Supplementary material for: Sequential Organ Failure Assessment (SOFA) score and quick SOFA(qSOFA) predict 30-day mortality in patients with HIV-associated Talaromycosis: A multicenter retrospective cohort study
Source: PLoS Negl Trop Dis. 2026 May 5;20(5):e0014278. doi: 10.1371/journal.pntd.0014278 (PMC13143057; doi:10.1371/journal.pntd.0014278)
Supplement: S1 File — (DOCX) [file pntd.0014278.s001.docx]

**S1.Process of cytokine and chemokine assay by FCM**

Interleukin (IL)-4, IL-6, IL-10, IL-17, tumor necrosis factor-alpha (TNF-α), and interferon-gamma (IFN-γ) levels were quantified using flow cytometry. Briefly, 50 μL of fresh plasma and 50 μL of reconstituted lyophilized cytokine standard mixtures were pipetted into a 96-well plate. Subsequently, magnetic beads conjugated with cytokine-specific capture antibodies (e.g., IL-6, TNF-α, IFN-γ) were added to each well. The plate was then sealed with a cover and incubated for 2 hours at room temperature (RT) on a plate shaker set at 300–500 rpm to facilitate binding. Following incubation, the plate was placed on a magnetic separator for 2 minutes to allow bead immobilization. The supernatant was carefully removed, and the wells were washed twice with wash buffer to eliminate unbound substances. Next, 200 μL of assay buffer was added to each well, and the beads were thoroughly resuspended by gentle pipetting or vortexing to ensure uniform distribution. The prepared samples were then analyzed using a flow cytometer equipped with the appropriate fluorescence detection channels. The assay had a dynamic detection range of 0–5,000 pg/mL.
