# Supplementary material for: Sequential Organ Failure Assessment (SOFA) score and quick SOFA(qSOFA) predict 30-day mortality in patients with HIV-associated Talaromycosis: A multicenter retrospective cohort study
Source: PLoS Negl Trop Dis. 2026 May 5;20(5):e0014278. doi: 10.1371/journal.pntd.0014278 (PMC13143057; doi:10.1371/journal.pntd.0014278)
Supplement: S2 File — (DOCX) [file pntd.0014278.s002.docx]

**S2. The criteria for assessment of the Sequential Organ Failure Assessment (SOFA) score and qSOFA**

1. **SOFA score**

| System | SOFA Score | | | | |
| --- | --- | --- | --- | --- | --- |
|  | 0 | 1 | 2 | 3 | 4 |
| Respiration |  |  |  |  |  |
| PaO_2_/FIO_2_, mm Hg | ≥400 | ＜400 | ＜300 | <200* | <100* |
| Coagulation |  |  |  |  |  |
| Platelets, ×10^9^/ L | ≥150 | ＜150 | <100 | <50 | <20 |
| Liver |  |  |  |  |  |
| Bilirubin, (µmol/L) | ＜20 | 20 – 32 | 33 – 101 | 102 – 204 | ＞204 |
| Cardiovascular | MAP ≥70 mm Hg | MAP＜70 mm Hg | Dop ≤5 or  Dob (any dose) † | Dop 5.1–15 or Epi ≤0.1  or Norepi≤0.1† | Dop >15 or  Epi >0.1  or Norepi >0.1† |
| Central nervous system | | | | | |
| Glasgow Coma Scale score | 15 | 13 – 14 | 10 –12 | 6 –9 | ＜6 |
| Renal |  |  |  |  |  |
| Creatinine, µmol/L | < 110 | 110 –170 | 171–299 | 300 – 440 | ＞440 |
| Urine output, mL/d |  |  |  | ＜500 | ＜200 |

Abbreviations: PaO_2_, partial pressure of oxygen; FIO_2_, fraction of inspired oxygen; MAP, mean arterial pressure; Dop, Dopamine; Dob, Dobutamine; Epi, Epinephrine; Norepi, Norepinephrine.

* Values are with respiratory support.

† The administration of catecholamines was maintained at doses measured in µg/kg/min for at least one hour.

1. **qSOFA score**

| **qSOFA indicator**  **(at admission)** | **Threshold** | **Point** |
| --- | --- | --- |
| Respiratory rate | ≥ 22 /min | 1 |
| Respiratory rate | Altered mental status (Glasgow Coma Scale < 15) | 1 |
| Systolic blood pressure | ≤ 100 mmHg | 1 |

Each abnormal parameter is scored with 1 point. Total qSOFA score ranges from 0 to 3.
